# Conversion effects of farmland to *Zanthoxylum bungeanum* plantations on soil organic carbon mineralization in the arid valley of the upper reaches of Yangtze River, China

**DOI:** 10.1371/journal.pone.0262961

**Published:** 2022-02-04

**Authors:** Chen Lv, Tahseen Saba, Jingyan Wang, Wenkai Hui, Wanlin Liu, Jiangtao Fan, Jiahui Wu, Xianzhi Liu, Wei Gong

**Affiliations:** Key Laboratory of National Forestry Administration on Forest Resources Conservation and Ecological Safety in the Upper Reaches of the Yangtze River, College of Forestry, Sichuan Agricultural University, Chengdu, China; Oakland University, UNITED STATES

## Abstract

Farmland conversion to forest is considered to be one of the effective measures to mitigate climate change. However, the impact of farmland conversion to forest land or grassland on soil CO_2_ emission in arid areas is unclear due to the lack of comparative information on soil organic carbon (SOC) mineralization of different conversion types. The SOC mineralization in 0–100 cm soil layer in farmland (FL), abandoned land (AL) and different ages (including 8, 15, 20 and 28 years) of *Zanthoxylum bungeanum* plantations were measured by laboratory incubation. The size and decomposition rate of fast pool (*C*_f_) and slow pool (*C*_s_) in different land-use types and soil layers were estimated by double exponential model. The results showed that: 1) Farmland conversion increased the cumulative CO_2_-C release (*C*_min_) and SOC mineralization efficiency, and those indexes in AL were higher than that in *Z*. *bungeanum* plantations. The *C*_min_ and SOC mineralization efficiency of 0–100 cm soil increased with the ages of *Z*. *bungeanum* plantation. Both *C*_min_ and SOC mineralization efficiency decreased with the increase of soil depth; 2) Both soil *C*_f_ and *C*_s_ increased after farmland converted to *Z*. *bungeanum* plantations and AL. The *C*_s_ in the same soil layer increased with the ages of *Z*. *bungeanum* plantation, and the *C*_f_ showed a “V” type with the increased ages of *Z*. *bungeanum* plantation. The *C*_f_ and *C*_s_ decreased with the increase of soil depth in all land-use types; 3) Farmland conversion increased the decomposition rate of *C*_f_ (*k*_1_) in all soil layer by 0.008–0.143 d^−1^ and 0.082–0.148 d^−1^ in *Z*. *bungeanum* plantations and AL, respectively. The *k*_1_ was obviously higher in the 0−20 cm soil layer than that in other soil layers, while the decomposition rate of *C*_s_ (*k*_2_) was not affected by FL conversion and soil depth; and 4) The initial soil chemical properties and enzyme activity affected SOC mineralization, especially the concentrations of total organic nitrogen (TON), SOC, easily oxidizable organic carbon (EOC) and microbial biomass carbon (MBC). It indicated that the conversion of farmland to *Z*. *bungeanum* plantations and AL increases SOC mineralization, especially in deeper soils, and it increased with the ages. The conversion of farmland to *Z*. *bungeanum* plantation is the optimal measure when the potential C sequestration of plant-soil system were taken in consideration.

## Introduction

The soil is the largest carbon (C) pool in terrestrial ecosystem, it stores about 1.5 × 10^15^ kg C [1, 2]. Small losses or sequestration in such a huge soil C pool will significantly change the soil-atmosphere C cycle and cause global climate change [3–5]. SOC mineralization is the main output flux of CO_2_ from soil to atmosphere, so it may be the key to regulate soil C pool, atmospheric CO_2_ concentration and global C cycle [6]. SOC mineralization and CO_2_ emission is mainly controlled by soil microorganisms [7], which directly depends on the quantity and stability of SOC, and is also affected by vegetation [8], hydrothermal conditions [9–11] and soil properties [12]. The conversion of farmland to forest land or grassland can usually improve SOC stocks by increasing C input (e.g. litter, roots and exudates). However, the increase of C input from soil C pool may also increase C output because it provides sufficient substrate for microorganisms and accelerates the mineralization of SOC [13–15]. In addition, farmland conversion would change the SOC mineralization matrix, and microbial and soil properties, which could significantly impact on the SOC mineralization process, and making the direction and degree of change in SOC mineralization uncertain [16, 17]. Therefore, the quantitative study of SOC mineralization and its driving factors after farmland conversion has become a hot spot in terrestrial ecosystem research.

SOC fractions are highly heterogeneous, it is important to study the size and decomposition rate of different SOC fractions pools for understanding SOC turnover [18, 19]. The dynamics model is widely used to distinguish different C pools because it is relatively simple and parameters are easy to obtain [20, 21]. Given the absence of exogenous organic matter in the incubation studies, it is typically considered reasonable to fit a mathematical model to the relationship between soil CO_2_ release and time in order to identify the size and decomposition rate of different C pools [22]. Previous studies have shown that the dynamics of SOC mineralization follows a two-phase pattern, including rapid decomposition in the early stage of incubation and stable decomposition in the middle and late stages. This is due to the low structural complexity of small-size labile organic matter, which are rapidly decomposed in the early stage of incubation [23]. After the consumption of small-size organics, the decomposition substances of microorganisms in the middle and later stages of incubation are mainly large-size organics that are difficult to decompose [24]. Therefore, SOC pools are usually divided into *C*_f_ and *C*_s_ according to turnover time, and the double exponential model is often used to estimate the size and decomposition rate of *C*_f_ and *C*_s_ [25, 26]. Recent studies found that long-term fertilization on Ultisol significantly promoted the size and decomposition rate of *C*_f_ and *C*_s_ [12]. The size and decomposition rate of *C*_f_ and *C*_s_ in different soil types and soil layers in subtropical zone are also different due to the degree of humification [23]. These studies have greatly improved our understanding of SOC mineralization process. However, the dynamic changes of SOC pool size and mineralization rate in different stable states after afforestation are still unclear [27, 28].

In previous studies, it was established that soil labile organic C (LOC) may significantly affect SOC mineralization in a short period of time [29, 30]. Among LOC, MBC is mainly composed of archaea, bacteria and eukaryotes, which is considered to be the most important factor controlling SOC mineralization [31]. However, other studies have found that MBC does not exert long-term control upon SOC mineralization [32]. EOC is mainly composed of amino acids, a small amount of MBC and other simple organic compounds, which may be an important part of the *C*_f_. It can be quickly decomposed and utilized by microorganisms, and have a significant impact on the SOC mineralization [33]. Soil pH and nitrogen (N) usually take part in the regulation of SOC mineralization by affecting microbial community composition and activity. The variation of soil CO_2_ emission with the increase of N concentration or pH in soils of different areas is not in consistent [34]. In addition, extractellular enzymes is another important factor affecting the mineralization of SOC. Some studies have shown that enzyme depolymerization limits the decomposition rate of soil organic matter (SOM). When the consumption rate of LOC is higher than that of recalcitrant organic C, the mineralization of SOC remains relatively stable [35, 36]. However, extractellular enzymes, while participating in the process of SOM decomposition into LOC, also release the nutrients needed by other microorganisms, which may stimulate or inhibit SOC mineralization. Generally, soil chemical and enzyme properties will affect SOC mineralization, but the direction and extent of the impact and the driving factors of SOC mineralization in different areas are still controversial.

*Zanthoxylum bungeanum* is an economic tree species with good economic and social value. At the same time, because of its strong drought tolerance, high adaptability to harsh habitats and fast growth, it has become one of the important tree species for the Chinese government in returning farmland to forest program in this area. At present, the planting area of *Z*. *bungeanum* in China alone is more than 4000 km^2^. However, there is almost no research on the ecological effects of *Z*. *bungeanum*, particularly information on the changes in SOC mineralization related to soil ecological functions. Thus, the objective of this study was to: 1) determine the direction and degree of SOC mineralization after farmland conversion; 2) estimate the changes in size, rate, and residence time of different soil C pools after farmland conversion; and 3) identify the main driving faction of SOC mineralization in soil properties of arid valleys. We hypothesized that the farmland conversion to forestland or grassland had a positive effect on SOC mineralization cumulative amount and efficiency; Soil *C*_s_ may also have a prominent contribution to SOC mineralization.

## Materials and methods

### Ethics statement

On behalf of, and having obtained permission from all the authors, I declare that: the paper is not currently being considered for publication elsewhere; all authors have been personally and actively involved in substantive work leading to the report, and will hold themselves jointly and individually responsible for its content. No specific permissions were required for these locations for soil sampling, and the field studies did not involve endangered or protected species.

### Study site

The study site is a *Z*. *bungeanum* cultivation base of Sichuan Agricultural University, located in Mao County, Aba Tibetan and Qiang Autonomous Prefecture, Sichuan, China (103°43′E, 30°51’N). This area is the transition zone between the Sichuan Basin and the eastern margin of the Qinghai-Tibet Plateau, with complex topography, and is a typical core area of the dry valley in the upper reaches of the Minjiang River. It belongs to the plateau monsoon climate, dry and windy, with obvious alternating dry and wet seasons. The annual average temperature is about 10–11°C, the annual accumulated temperature ≥10°C is 4 071.5°C, and the annual precipitation is about 490 mm, mainly in June-September [37]. The soil is yellowish brown in color and is classified as Haplastepts in the USDA (American soil classification system). The vegetation types in the arid valley are mainly mesophyte drought-tolerant plants, which adapt to the dry valley climate.

Three land-use types including farmland, abandoned land and different age of *Z*. *bungeanum* plantations were selected for the study (Table 1). According to the local traditional farming habits, the main crops of FL are potato and corn. AL is a naturally formed grassland (28 years) after the abandonment of farmland. Most of the plants are annual herbs, mainly including *Artemisia carvifolia*, *Lycium chinense*, *Rubia cordifolia*, *Vicia sepium*, *Stellaria media*, *Trigonotis peduncularis*, and *Medicago sativa*, etc. The planting years of *Z*. *bungeanum* plantations are 8-year (ZB8), 15-year (ZB15), 20-year (ZB20) and 28-year (ZB28), and all of them keep similar and traditional local management practices. *Z*. *bungeanum* plants were spaced 3 m × 4 m and fertilized with 125 kg N ha^–1^ yr^–1^, 60 kg P_2_O_5_ ha^–1^ yr^–1^ and 60 kg K_2_O ha^–1^ yr^–1^.

**Table 1:** Geographical and plant community characteristics of farmland conversion sites in Arid Valley.

Code	Altitude (m)	Slope (°)	Aspect	Tree height (m)	Ground diameter (cm)	Canopy density (%)	Soil type	Temperature (°C)
**FL**	2647	23	SW137°	—	—	—	Haplustepts	10.1
**ZB8**	2663	22	SW138°	2.46±0.27	7.9±0.74	74.1±1.02	Haplustepts	10.2
**ZB15**	2655	21	SW123°	2.93±0.36	14.1±1.02	79.3±0.85	Haplustepts	10.6
**ZB20**	2660	24	SW140°	3.24±0.23	19.4±1.75	81.7±1.06	Haplustepts	10.5
**ZB28**	2652	23	SW131°	3.26±0.39	24.5±2.19	85.5±0.99	Haplustepts	10.6
**AL**	2649	23	SW129°	0.81±0.17	—	65.3±0.08	Haplustepts	10.9

The tree height and canopy density data of AL are the height and coverage of grass, respectively. The temperature is the annual average temperature.

### Soil sampling

In January 2020, three plots (10 m × 10 m) with the same soil type and similar altitude, aspect and slope gradients were selected for every land-use types (including FL, AL, ZB8, ZB15, ZB20 and ZB28). The litters were removed before sampling. Five soil samples were randomly collected in each plot using a soil coring tool (5.0 cm in diameter, height 20 cm) in the same soil horizons of 0–20 cm, 20–40 cm, 40–60 cm, 60–80 cm and 80–100 cm. After sampling, five samples of the same soil layer were combined into a composite sample of each plot. After roots and other plant debris were removed, the samples were homogenized and divided into two parts. One part was sieved through a 2-mm mesh immediately and used for the determination of soil moisture content (SWC), MBC and SOC mineralization. The other part was air-dried and sieved through a 2-mm mesh. Subsamples of < 2 mm soil were then ground with a porcelain mortar for determination of soil chemical properties and enzyme activities.

### Laboratory measurements

Fresh soil samples were dried at 105°C to determine the SWC. Total SOC was determined by the dichromate wet oxidation method [38]. Soil pH, concentration of TON were measured as described by Chen et al. [39] and Lu et al. [40], respectively. The activities of urease (URE) and invertase (INV) were measured as described by Doran [41]. MBC was measured by the chloroform fumigation extraction method [42]. EOC was measured as described by Blair et al. [43].

For the determination of SOC mineralization, 50 g of bulk soils at 60% field water capacity (FWC) along with 25-mL glass vial containing 20 mL of 0.2 M NaOH to trap the released CO_2_ were incubated in the dark at 25°C in 250-mL jars. Three control jars containing NaOH without soil were also prepared. Samples were preincubated for 5 d to remove the flush of CO_2_ caused by re-wetting. Soil moisture was maintained throughout incubation by adding de-ionized water. CO_2_ captured, was measured once on days 1, 3, 7, 14, 21, 28, 35 and 42 after incubation, and 0.1 M HCl was used as titrant. The *C*_min_ (mg CO_2_-C kg^−1^ soil) was calculated by summing the CO_2_-C released during each incubation period [44].

The double exponential model [25] was used to estimate SOC mineralization kinetics. The double exponential model can evaluate the size and decomposition rate of labile and recalcitrant pools.

$$C_{t}=C_{f}(1‐\exp(‐k_{1}\times t))+C_{s}(1‐\exp(‐k_{2}\times t))$$ (1)

where *C*_t_ is the cumulative mineralized SOC (mg CO_2_-C kg^−1^) at time t (d); *C*_f_ and *C*_s_ represent SOC content (mg CO_2_-C kg^−1^) of the fast and slow pools, respectively; *k*_1_ and *k*_2_ represent decomposition rate constants (d^−1^) of the *C*_f_ and *C*_s_, respectively; *t*_1_ and *t*_2_ are the residence time (d) of *C*_f_ and *C*_s_, and are reciprocal related to the *k*_1_ and *k*_2_, respectively.

SOC mineralization efficiency (%) is calculated by the following formula:

$$\mathrm{SOC}\;\mathrm{mineralization}\;\mathrm{efficiency}=\frac{C_{\min}}{\mathrm{SOC}\times 1000}\times 100\%$$ (2)

### Statistical analysis

Excel 2010 and SPSS 20.0 software were used for fitting and statistical analysis and Origin 2018 was used for drawing. Two-way ANOVA was run on different soil chemistry and enzyme properties with sites and depths as fixed variables. Before the analysis, all data were standardized in order to improve the uniformity and normality of variance.

Since there is no exogenous organic matter added during soil incubation, the relationship among soil chemistry, enzyme parameters and SOC mineralization can be found by evaluating the relative importance of soil initial chemistry and enzyme parameters before incubation. However, soil chemistry and enzyme parameters are usually autocorrelated. Therefore, partial least squares regression (PLS-R) model was used to explain the effects of soil chemistry and enzyme parameters on the *C*_min_, SOC mineralization efficiency, *C*_f_, *C*_s_ and *k*_1_, because PLS-R eliminated multicollinearity among predictors by linear transformation from a large number of predictors to a small number of orthogonal factors. The variable importance of projection (VIP) value can be used to estimate the relative importance of chemistry and enzyme parameters, and PLS-R coefficient can be used to explain the influence degree and direction of chemistry and enzyme parameters on SOC mineralization.

## Results

### Mineralization of SOC

**Fig 1:**
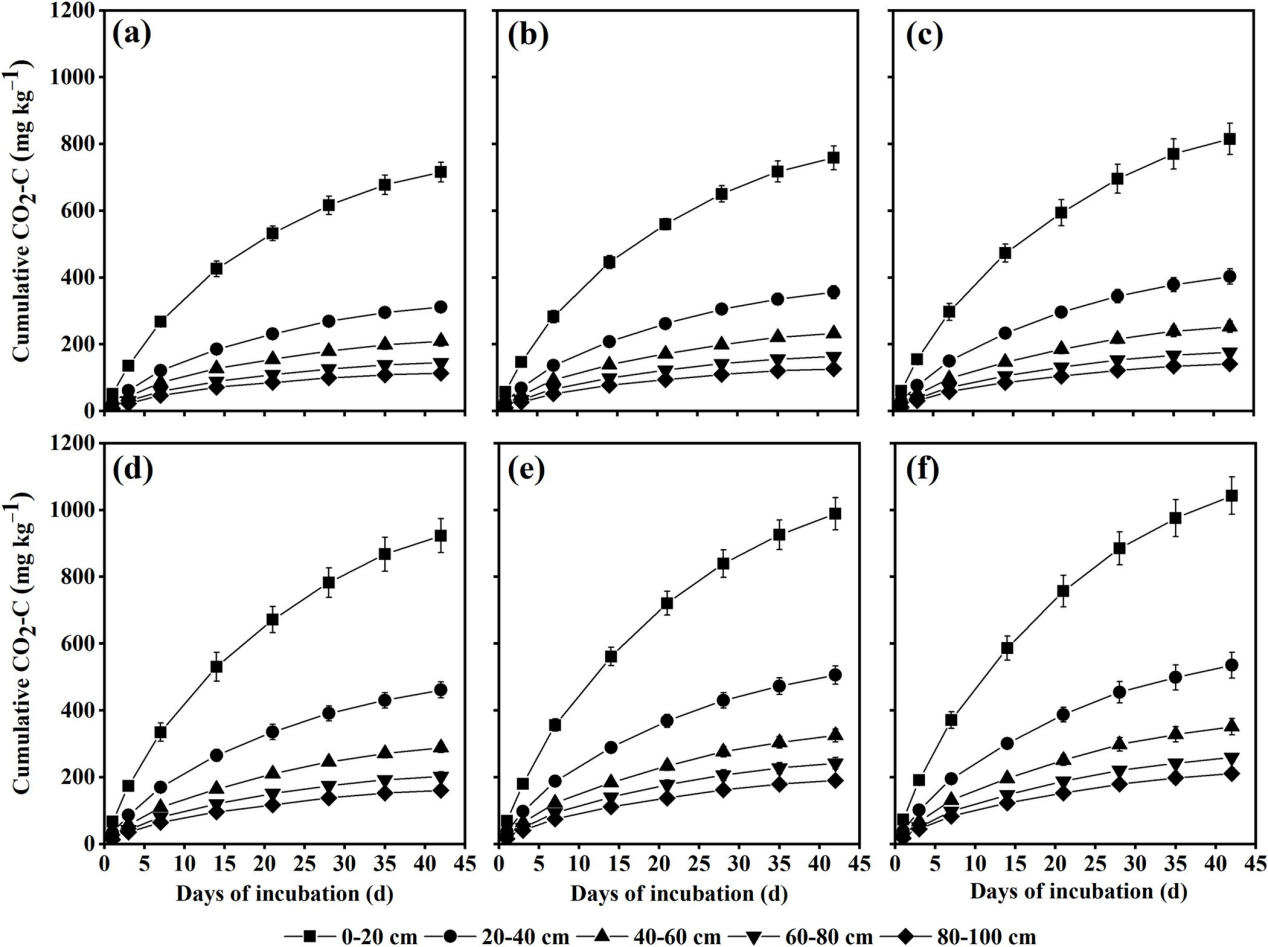
Cumulative CO_2_ released process (mg CO_2_-C kg^−1^ soil) from *Z*. *bungeanum* plantations in Arid Valley in soils over the incubation period. (a): FL; (b): ZB8; (c): ZB15; (d): ZB20; (e): ZB28; (f): AL. Error bars represent standard deviations (*n* = 3).

During the 42-day incubation period, the SOC mineralization process was simulated at 0−100 cm depth in all land-use types, and it was found that the cumulative SOC mineralization increased with the incubation time (Fig 1). The daily SOC mineralization rate in all soil layers of the land-use types was the highest in the early stage of incubation (day 1), and then decreased with the incubation time. The daily SOC mineralization rate of 0−20 cm soil layer in the early incubation stage in FL, ZB8, ZB15, ZB20, ZB28 and AL was 51.8, 57.6, 60.8, 67.2, 69.8 and 73.8 mg kg^−1^ d^−1^, respectively, which is higher than that of other soil layers.

In each stage of the whole incubation period, the CO_2_ emissions of each soil layer in AL and *Z*. *bungeanum* plantations were higher than FL, especially AL was found to be the highest. The CO_2_ emission of same soil layer increased with the ages of *Z*. *bungeanum* plantation in each stage of the whole incubation period, and in which the soil CO_2_ emissions of all land-use types decreased with the increase of soil depth.

**Fig 2:**
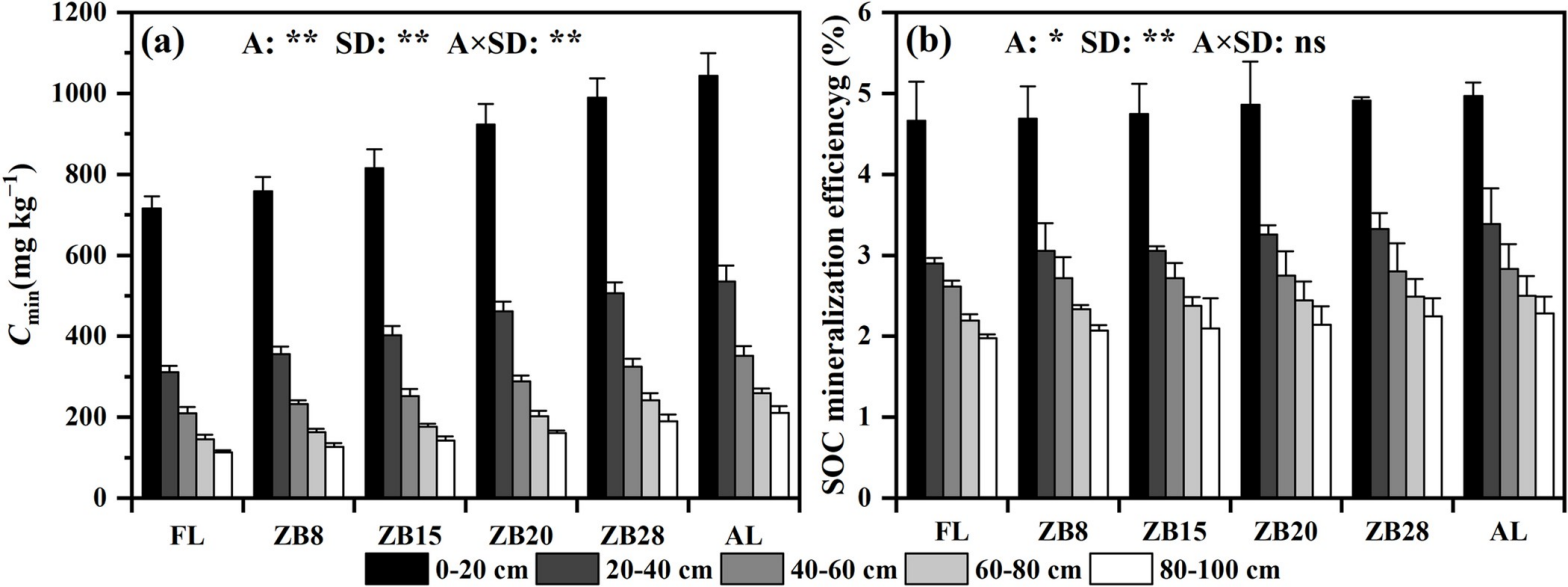
*C*_min_ (a) and SOC mineralization efficiency (b) for farmland conversion in Arid Valley in soils over the incubation period. Error bars represent standard deviations (*n* = 3). Significant differences for main effects of sites (A), soil depth (SD) and their interactions are shown at *: *p* < 0.05, **: *p* < 0.01, ns: *p* > 0.05.

Sites significantly affected *C*_min_ (*p* < 0.01) and SOC mineralization efficiency (*p* < 0.05), and both of them were significantly affected by soil depth (*p* < 0.01). The interaction between sites and soil depth significantly affected *C*_min_ (*p* < 0.01). After 42 days of incubation, the *C*_min_ of FL, *Z*. *bungeanum* plantations (including ZB8, ZB15, ZB20 and ZB28) and AL were 113.7–715.8 mg kg^−1^, 126.4–988.9 mg kg^−1^ and 210.7–1043.1 mg kg^−1^, respectively (Fig 2A). The SOC mineralization efficiency of the same soil layer among different types was the highest in AL and the lowest in FL (Fig 2B). Both *C*_min_ and SOC mineralization efficiency increased with the ages of *Z*. *bungeanum* plantation in all soil layer, and decreased with the increasing soil depth in all land-use types. The *C*_min_ increased by 5.9%–45.7% and 11.2%–85.3% in 0−20 cm and 80−100 cm soil layers, respectively, after FL conversion to *Z*. *bungeanum* plantations and AL.

### Estimation of SOC mineralization parameters

SOC mineralization parameters were estimated by double exponential model as shown in Table 2. The double exponential model fitted the SOC mineralization process well (*R*^2^ > 0.999). The pool was estimated by using the double exponential model, the results showed that the size of *C*_f_ and *C*_s_ decreased with the increase of soil depth in all land-use types. The size of the *C*_f_ ranged from 24.5 to 91.7 mg CO_2_-C kg^−1^. The size of *C*_f_ in almost all soil layers of AL was higher than that of FL. After FL was converted to *Z*. *bungeanum* plantations, the size of *C*_f_ in all soil layers decreased in the early stage of planting, but increased rapidly in the later stages. The size of the *C*_s_ ranged from 106.8 to 1178.0 mg CO_2_-C kg^−1^. In the three land-use types, the size of *C*_s_ of the same soil layer is the highest in AL and the lowest in FL. The size of *C*_s_ in the same soil layer increased with the ages of *Z*. *bungeanum* plantation. After FL conversion to *Z*. *bungeanum* plantations or AL, the size of *C*s in 0−20 cm soil layer and 80−100 cm soil layer increased by 7.9%–54.9% and 20.2%–115.4%, respectively.

**Table 2:** Parameter estimation of double exponential model for farmland conversion in Arid Valley.

Sites	Soil depth (cm)	*C*_f_	*C*_s_	*k*_1_	*k*_2_	*t*_1_	*t*_2_	*R* ^2^
**FL**	**0–20**	77.86	760.4	0.272	0.044	3.68	22.79	1.000
	**20–40**	44.90	326.3	0.267	0.041	3.74	24.40	1.000
	**40–60**	49.89	212.5	0.222	0.034	4.50	29.68	0.999
	**60–80**	24.60	146.9	0.280	0.042	3.58	23.93	0.999
	**80–100**	29.32	106.8	0.188	0.038	5.32	26.43	0.999
**ZB8**	**0–20**	75.85	820.4	0.349	0.043	2.86	23.31	1.000
	**20–40**	46.79	381.9	0.299	0.040	3.34	25.08	1.000
	**40–60**	45.06	243.5	0.258	0.036	3.87	28.02	0.999
	**60–80**	24.51	168.1	0.320	0.043	3.12	23.48	0.999
	**80–100**	24.62	128.4	0.265	0.038	3.78	26.02	0.999
**ZB15**	**0–20**	73.61	901.9	0.379	0.042	2.64	24.03	1.000
	**20–40**	42.37	437.9	0.326	0.041	3.07	24.11	1.000
	**40–60**	33.67	273.6	0.333	0.039	3.01	25.67	1.000
	**60–80**	25.15	184.5	0.358	0.042	2.79	24.07	0.999
	**80–100**	29.29	147.5	0.302	0.035	3.32	28.64	0.999
**ZB20**	**0–20**	91.72	1023.9	0.345	0.040	2.90	24.95	1.000
	**20–40**	59.88	505.4	0.275	0.038	3.64	26.48	1.000
	**40–60**	37.59	318.1	0.355	0.037	2.82	26.69	0.999
	**60–80**	29.27	212.9	0.341	0.041	2.93	24.50	1.000
	**80–100**	29.11	170.9	0.331	0.036	3.02	28.01	0.999
**ZB28**	**0–20**	84.42	1103.5	0.352	0.041	2.84	24.40	1.000
	**20–40**	57.77	558.1	0.345	0.039	2.90	25.72	1.000
	**40–60**	42.19	364.2	0.360	0.036	2.78	27.70	0.999
	**60–80**	34.57	260.9	0.333	0.038	3.01	26.23	1.000
	**80–100**	35.36	208.0	0.315	0.033	3.18	30.27	1.000
**AL**	**0–20**	78.47	1178.0	0.420	0.041	2.38	24.46	1.000
	**20–40**	53.40	600.8	0.384	0.039	2.60	25.81	1.000
	**40–60**	46.39	400.5	0.338	0.035	2.96	28.92	0.999
	**60–80**	31.76	286.6	0.362	0.038	2.76	26.45	1.000
	**80–100**	36.74	230.0	0.335	0.034	2.99	29.34	1.000

The estimation results of double exponential model showed that *k*_1_ and *k*_2_ of 0−20 cm soil layer are larger than those of other soil layers (Table 2). After FL was converted to AL and *Z*. *bungeanum* plantations, the *k*_1_ of all soil layers was increased, especially in AL. The *k*_1_ in all soil layers increased rapidly in the early stage of *Z*. *bungeanum* planting, but it did not increase in the later stage. The *k*_2_ in the same soil layer is almost similar among different land-use types. The *t*_1_ and *t*_2_ of 0−20 cm soil layer are the smallest compared with other soil layers (Table 2). The *t*_1_ decreased by 0.82–2.33 d and 0.10–2.30 d after farmland was converted to AL and *Z*. *bungeanum* plantations, respectively. The *t*_2_ of FL, *Z*. *bungeanum* plantations (including ZB8, ZB15, ZB20 and ZB28) and AL were 22.8–29.7 d, 23.3–30.3 d and 24.5–29.3 d, respectively.

### Chemistry and enzyme parameters of soil before SOC mineralization

**Fig 3:**
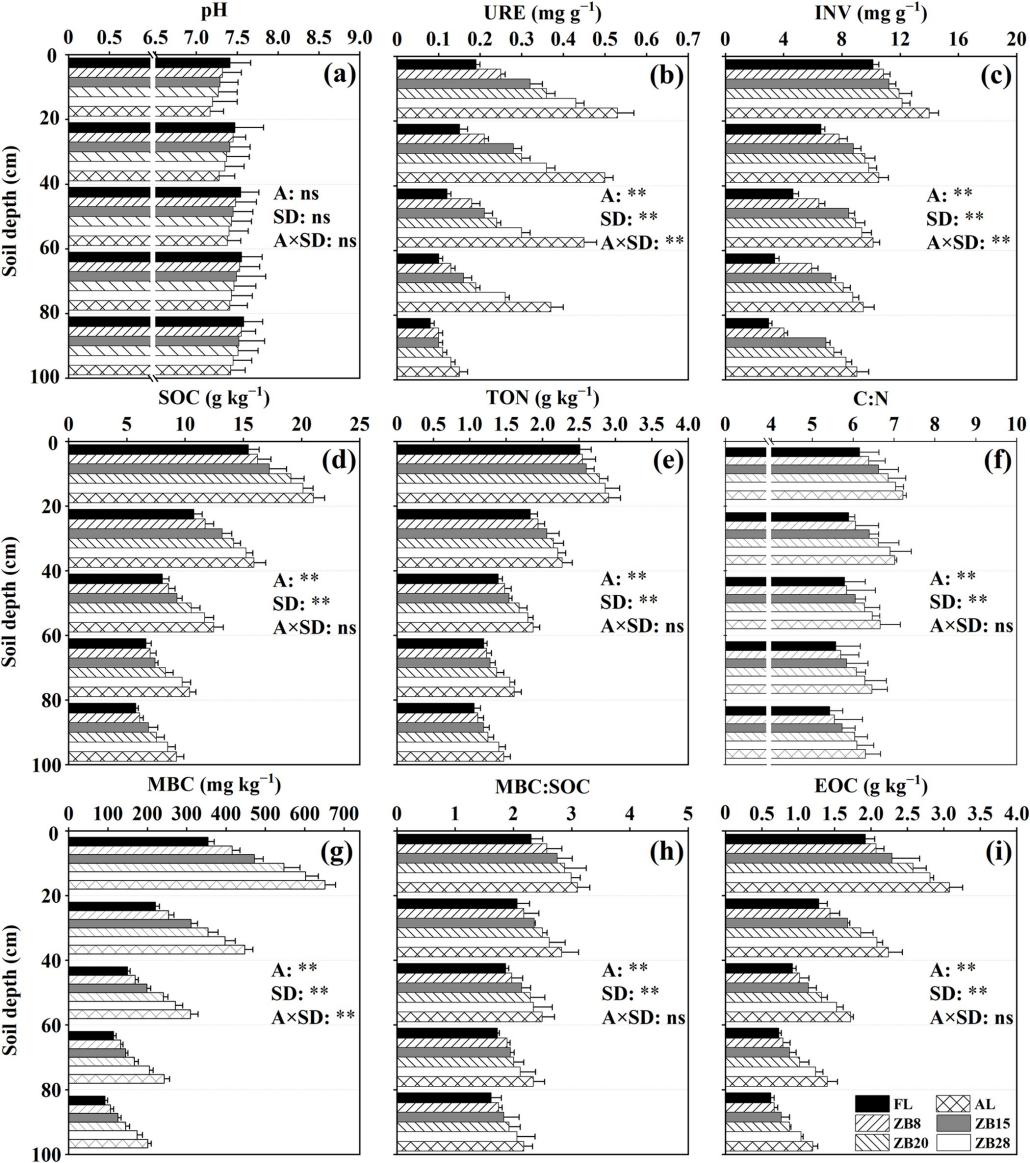
Basic chemistry and enzyme properties of soil in different years of returning farmland. Error bars represent standard deviations (*n* = 3). Significant differences for main effects of sites (A), soil depth (SD) and their interactions are shown at *: *p* < 0.05, **: *p* < 0.01, ns: *p* > 0.05.

The results of Two-way ANOVA showed that sites and soil depth had significant effects on soil C/N, MBC/SOC and URE, INV, SOC, TON, MBC and EOC concentrations (*p* < 0.01, Fig 3B–3I). Soil URE, INV and MBC were significantly affected by the interaction between sites and soil depth (*p* < 0.01). The lowest and highest soil pH of the same soil layer among different land-use types is in AL and FL, respectively (Fig 3A). Soil pH in the same soil layer decreased with the ages of *Z*. *bungeanum* plantation. Soil pH increased with increasing soil depth in all land-use types. After FL was converted to AL and *Z*. *bungeanum* plantations, the C/N, MBC/SOC values and URE, INV, SOC, TON, MBC, and EOC concentrations of all soil layers were increased, especially in AL (Fig 3B–3I). The C/N, MBC/SOC values and URE, INV, SOC, TON, MBC, and EOC concentrations in the same soil layer increased with the age of *Z*. *bungeanum* plantation. The C/N and MBC/SOC, and URE, INV, SOC, TON, MBC and EOC concentrations of soils decreased with the soil depth in all land-use types.

### Factors driving SOC mineralization

**Fig 4:**
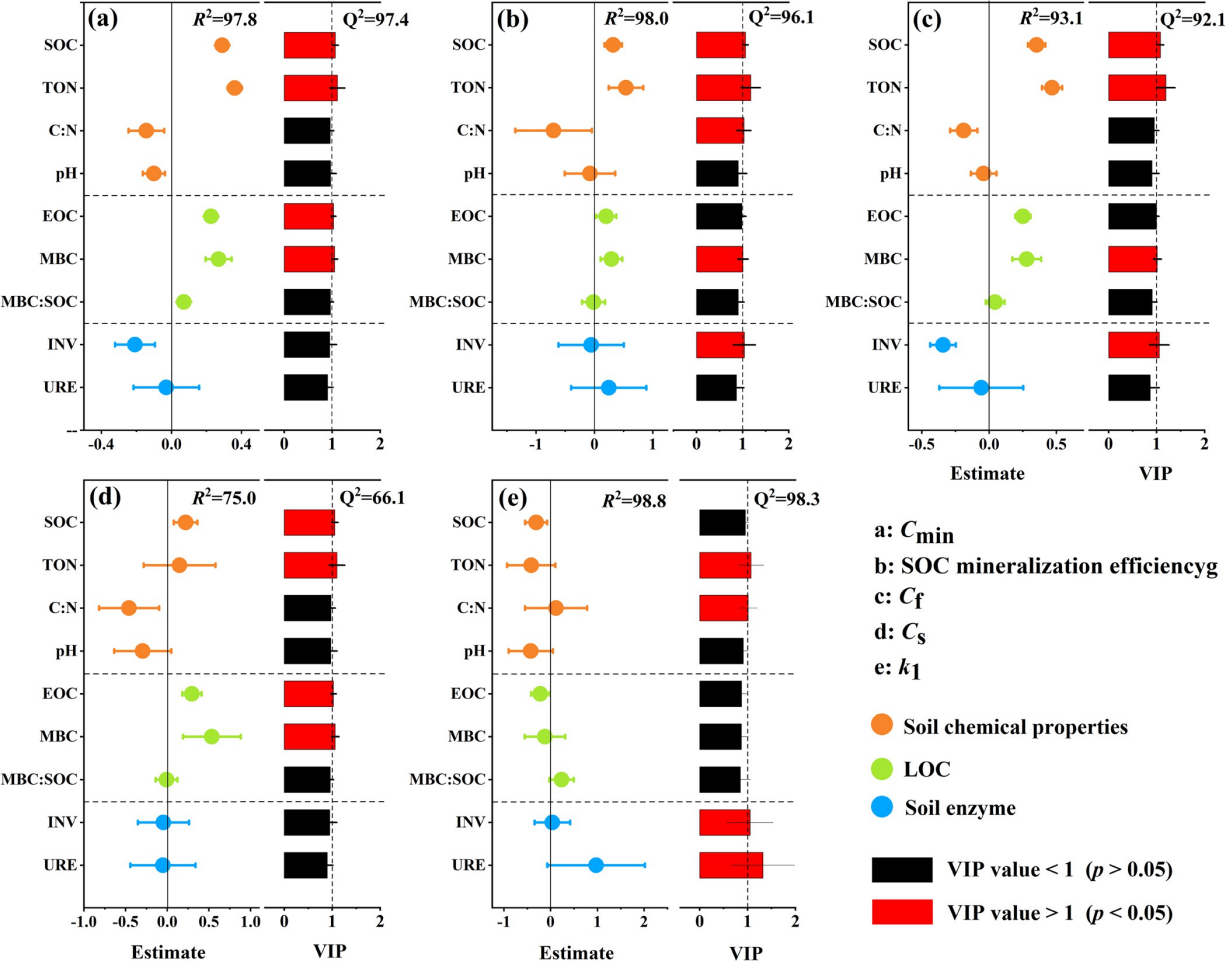
The effects of soil initial chemistry and enzyme parameters on the *C*_min_ (a), SOC minimization efficiency (b), size of *C*_f_ (c), size of *C*_s_ (d) and *k*_1_ (e) were studied by partial least squares model. The estimation indicates the degree and direction of SOC mineralization affected by initial soil chemical parameters. The parameter types are distinguished by different colors. When the VIP value of each parameter is greater than 1 (red), it indicates that it has potential significance (*p* < 0.05). The error bars represent the confidence intervals at the 95% level.

The initial chemical and enzyme parameters of soil explained 75.0%–98.8% of the mineralization parameters of SOC, which greatly drove the mineralization process of SOC (Fig 4). The degree and direction of each SOC mineralization parameters are affected by specific soil chemical and enzyme parameters. The establishment of *k*_2_ model was abandoned because the initial chemistry and enzyme parameters of soil could only explain 4.9% of *k*_2_ information. Higher concentrations of SOC, TON, EOC and MBC significantly promoted *C*_min_ (Fig 4A) and *C*_s_ (Fig 4D). Initial concentrations of SOC, TON and MBC significantly increased the mineralization efficiency of SOC, but were significantly inhibited by high C/N ratio and INV concentration (Fig 4B). The *C*_f_ was significantly controlled by the increase of initial SOC, TON and MBC concentration, but limited by high INV concentration (Fig 4C). The decomposition *k*_1_ is limited by high C/N value and TON concentration, and the *k*_1_ is accelerated by high INV and URE concentration (Fig 4E).

## Discussion

SOC mineralization affects the production of CO_2_ and nutrient availability in soil, reflecting the turnover and stability of SOC [6, 15]. In our study, the *C*_min_ of the same soil layer increases with the planting age of *Z*. *bungeanum*, and the conversion of farmland to AL has a higher *C*_min_ than *Z*. *bungeanum* plantation. This may be because the C input of soil increases with the age of planting trees, and the C input of annual herbs in AL may be higher than that of *Z*. *bungeanum* plantation with a large number of fruits harvested. The increase of C input provides more SOC mineralized matrix [45, 46]. Wang et al. [21] also found that SOC mineralization increased with the age of *Robinia pseudoacacia* plantation. However, the report of Zhu et al. [34] shows that the conversion of farmland to tea plantation in Western Sichuan reduces *C*_min_, and the *C*_min_ of tea plantation is also lower than that of *Z*. *bungeanum* plantation. The litter produced by tea plantation of evergreen shrub is usually lower than that of *Z*. *bungeanum* plantation of deciduous shrub, which means there is less substrate. In addition, the acidic environment and the decrease of N concentration in the soil of tea plantation are not conducive to microbial growth, which may jointly inhibit SOC mineralization [47]. Our study also found that deeper soil has a high *C*_min_ change rate. Deep soil microorganisms in C-poor environment are usually more sensitive to C input. Therefore, when root exudates that can be directly used by organisms are input in deep soil, microorganisms may accelerate the decomposition of *C*_s_ due to "priming effect" [48–50].

The size and decomposition rate of the different pools of SOC are critical in determining whether the soil is a C source or sink [51]. *C*_f_ are generally considered to determine SOC mineralization, but Wang et al. [21] found that CO_2_ emissions from slow and passive pools accounted for more than 50% of total CO_2_ emissions. In this study, the size of *C*_s_ is much higher than *C*_f_. Although the *C*_s_ has a long turnover time, it still has an important contribution to SOC mineralization due to its large size [52]. Our results show that the size of *C*_f_ decreased in the early stage of *Z*. *bungeanum* planting and increased rapidly in the later stages, while the size of *C*_s_ increased continuously with the age of *Z*. *bungeanum* plantation. This may be that less C input in the early stage of afforestation is not enough for rapidly changing microorganisms, resulting in the reduction of rapidly decomposable *C*_f_. In the later stage of afforestation, the number of microorganisms remained unchanged due to environmental constraints, and more C input expanded the size of *C*_f_ after compensating the loss caused by SOC mineralization. The continuous increase of *C*_s_ may be that the C input is greater than its slower decomposition. Our study found that the *k*_1_ and *k*_2_ in the arid valley were 0.188–0.420 d^−1^ and 0.033–0.044 d^−1^, respectively. Xu et al. [12] reported that the *k*_1_ and *k*_2_ on Ultisol were 0.049–0.079 d^−1^ and 0.0006–0.0019 d^−1^, while Yang et al. [26] found that the *k*_1_ and *k*_2_ in different forest zones of China were 0.023–0.125 d^−1^ and < 0.0001 d^−1^, respectively. This indicates that the decomposition of SOC in arid valley area may be stronger than that in other areas.

Using the least squares model estimation, it was found that high C/N, INV and URE concentrations accelerated *k*_1_, while high TON concentrations limited *k*_1_ (Fig 4E). The speed of microbial driven SOC mineralization is affected by substrate C/N [53, 54]. The C/N of microorganisms is about 5:1, which means that when microorganisms decompose organic matter, they need to assimilate 5 parts of C and 1 part of N to form cell body. In addition, assimilating 1 part of C needs to consume 4 parts of C to obtain energy. Therefore, the optimal C/N for substrate decomposition is about 25:1. In our study, soil C/N is about 5–8, which is far lower than the optimal C/N of substrate decomposition. When soil C/N increases, it will accelerate *k*_1_ because it is closer to the optimal C/N. The optimal C/N also highlights the importance of TON, because the increase of TON means that more SOC increase is required to achieve the optimal C/N, otherwise the input imbalance between C and N will limit *k*_1_. The transformation and decomposition of organic matter are inseparable from enzymes. SOM with complex structure is first degraded under the catalysis of enzymes to obtain LOC with simple structure, which will decompose rapidly again with the participation of enzymes [35]. Therefore, high INV and URE concentrations may change the quantity and quality of microbial degradation matrix, thus accelerating *k*_1_.

It is not surprising that *C*_s_ and *C*_f_ are significantly affected by SOC and MBC, because SOC and MBC are important parts of C pool. It is worth mentioning that *C*_min_, SOC mineralization efficiency, *C*_f_, *C*_s_ and *k*_1_ are significantly affected by TON. N limitation was most obvious in the arid valley where soil nutrients were relatively low. The decomposition of plant residues by microorganisms usually involves the assimilation of N in soil, and the process of extracellular enzyme secretion by microorganisms may also be controlled by N [55]. Jiang et al. [23] compared the changes of C and N before and after incubation and their correlation with SOC mineralization, and found that the availability of N was more important limiting factor for SOC mineralization than C. At the same time, some studies also found that the increase of SOC mineralization was consistent with the increase of N [56]. Interestingly, high INV concentration limits the mineralization efficiency of *C*_f_ and SOC, which may be due to the involvement of INV in soil N transformation, which inhibits the mineralization efficiency of *C*_f_ and SOC by changing N concentration.

Previous studies have shown that the role of enzyme activity in SOC mineralization is greater than that in C pool, because C should be degraded by enzymes before it can be directly absorbed by microorganisms [12]. Current study found that high concentrations of SOC, TON, EOC and MBC significantly promoted *C*_min_, which is usually determined by the size and decomposition rate of the C pool. Results showed that INV and URE are the main factors affecting the decomposition rate of C pool, while SOC, EOC and MBC affect both C pools and *C*_min_. This indicates that the change of *C*_min_ in arid valley is mainly determined by the size of the pool rather than the decomposition rate, and that the direct effects of soil matrix on *C*_min_ is less mediated by enzyme. *C*_s_ and *C*_f_ reflect the quantity and quality of SOC respectively, which affect the turnover of SOC and are closely related to SOC mineralization [57]. Xie et al. [58] found that the high content of SOC promoted the mineralization of SOC. This imply that the matrix content of SOC is high, which is convenient for microorganisms to use and produce more CO_2_ [23, 59].

## Conclusions

Farmland conversion to forestland or abandoned land increased *C*_min_, SOC mineralization efficiency, *C*_s_ and *k*_1_, and those indexes in AL were higher than that of *Z*. *bungeanum* plantations. The *C*_min_, SOC mineralization efficiency and *C*_s_ increased with the age of *Z*. *bungeanum* plantations. The *C*_f_ decreased in the initial stage of *Z*. *bungeanum* afforestation, but it increased rapidly in the later stages. The *C*_min_, SOC mineralization efficiency, *C*_f_ and *C*_s_ decreased with the increase of soil depth, and *k*_1_ in 0–20 cm soil layer were higher than those in deeper soil layers. However, sites and soil depth had no obvious impacts on *k*_2_. The *C*_min_ is strongly driven by initial concentrations of TON, SOC, EOC and MBC, and especially N is the most important limiting factor of SOC mineralization in arid valley areas with relatively low soil nutrients.

## Supporting information

S1 Dataset (DOCX)
