# Supplementary material for: Conversion effects of farmland to Zanthoxylum bungeanum plantations on soil organic carbon mineralization in the arid valley of the upper reaches of Yangtze River, China
Source: PLoS One. 2022 Feb 4;17(2):e0262961. doi: 10.1371/journal.pone.0262961 (PMC8815984; doi:10.1371/journal.pone.0262961)
Supplement: S1 Dataset — (DOCX) [file pone.0262961.s001.docx]

**S1. Cumulative CO_2_ released process (mg CO_2_-C kg^−1^ soil) from each land-use types over the 42-day study (Mean ± SD)**

| **Code** | **Soil depth** | **Cumulative CO_2_-C (mg CO_2_-C kg^−1^ soil)** | | | | | | | |
| --- | --- | --- | --- | --- | --- | --- | --- | --- | --- |
|  |  | **1 th** | **3 th** | **7 th** | **14 th** | **21 th** | **28 th** | **35 th** | **42 th** |
| **ZB28** | **0-20cm** | 69.8±3.4 | 180.6±7.7 | 356.4±18.5 | 561.6±27.7 | 721.1±35.3 | 839.7±40.8 | 926.2±44.4 | 988.9±48.6 |
| **ZB28** | **20-40cm** | 37.9±1.5 | 97.8±4.7 | 187.9±8.2 | 289.2±13.6 | 368.7±19.2 | 430.1±22.7 | 472.6±24.8 | 506.1±27.3 |
| **ZB28** | **40-60cm** | 25.2±1.1 | 64.3±2.6 | 122.9±7.0 | 183.7±10.8 | 234.4±14.2 | 276.6±15.8 | 304.4±17.4 | 324.9±19.2 |
| **ZB28** | **60-80cm** | 19.4±1.8 | 49.3±4.1 | 94.0±7.9 | 140.6±11.5 | 177.9±14.3 | 206.8±15.7 | 227.5±16.5 | 242.1±16.9 |
| **ZB28** | **80-100cm** | 16.2±2.4 | 40.7±4.5 | 75.3±7.4 | 111.9±8.9 | 137.7±11.6 | 161.9±14.3 | 179.2±15.4 | 190.4±16.2 |
| **ZB20** | **0-20cm** | 67.2±3.9 | 174.5±12.9 | 335.0±27.7 | 530.9±43.1 | 671.9±38.9 | 782.7±43.9 | 867.5±50.5 | 923.2±50.7 |
| **ZB20** | **20-40cm** | 33.7±2.7 | 86.5±4.6 | 169.9±9.6 | 265.4±18.1 | 335.5±22.8 | 391.1±22.4 | 430.2±22.7 | 461.5±24.3 |
| **ZB20** | **40-60cm** | 22.6±2.3 | 57.5±5.7 | 110.4±6.3 | 164.7±11.4 | 210.2±12.5 | 246.1±11.6 | 271.3±13.6 | 288.4±14.7 |
| **ZB20** | **60-80cm** | 16.7±1.4 | 42.9±2.6 | 80.8±4.2 | 120.5±8.0 | 151.5±8.9 | 174.9±11.0 | 192.3±12.0 | 202.9±13.0 |
| **ZB20** | **80-100cm** | 14.1±2.1 | 35.4±2.1 | 64.7±2.2 | 96.2±2.9 | 117.5±4.0 | 138.1±5.4 | 152.4±6.4 | 160.8±6.8 |
| **ZB15** | **0-20cm** | 60.8±6.5 | 154.7±12.6 | 297.2±25.3 | 472.9±26.8 | 594.1±39.0 | 695.6±42.9 | 770.3±45.3 | 815.0±47.0 |
| **ZB15** | **20-40cm** | 29.9±2.6 | 76.8±5.6 | 149.6±11.3 | 233.9±12.9 | 296.4±14.9 | 344.0±20.1 | 378.3±20.8 | 402.9±22.7 |
| **ZB15** | **40-60cm** | 20±1.1 | 50.4±1.6 | 97.6±6.4 | 146.9±10.8 | 185.5±13.7 | 216.3±14.2 | 239.1±16.0 | 252.5±17.0 |
| **ZB15** | **60-80cm** | 14.8±0.7 | 37.7±1.7 | 70.9±4.5 | 105.3±7.0 | 131.6±6.6 | 153.0±7.2 | 167.9±7.6 | 176.2±8.0 |
| **ZB15** | **80-100cm** | 12.6±1.0 | 31.7±1.9 | 58.4±3.9 | 86.0±6.5 | 104.5±8.3 | 121.9±9.8 | 134.9±10.8 | 141.8±10.8 |
| **ZB8** | **0-20cm** | 57.6±2.7 | 147.2±7.9 | 283.2±18.3 | 446.2±19.3 | 559.9±17 | 650.2±24.3 | 717.3±31.5 | 758.3±35.3 |
| **ZB8** | **20-40cm** | 27.2±0.3 | 69.2±4.3 | 136.6±6.4 | 207.7±6.3 | 262±11.2 | 305.5±15.2 | 335.1±17.3 | 356.0±18.9 |
| **ZB8** | **40-60cm** | 19.0±2.0 | 47.7±1.7 | 93.2±3.8 | 139.1±6.2 | 171.8±5.3 | 199.2±7.6 | 220.9±8.3 | 232.8±9.4 |
| **ZB8** | **60-80cm** | 13.7±1.4 | 34.6±2.9 | 66.4±6.0 | 99.1±7.0 | 122.7±7.0 | 142.5±7.2 | 155.9±7.5 | 163.4±8.0 |
| **ZB8** | **80-100cm** | 10.6±1.1 | 26.9±2.4 | 51.8±5.1 | 77.5±5.3 | 94.3±6.9 | 109.6±7.8 | 121.0±8.8 | 126.4±9.3 |
| **FL** | **0-20cm** | 51.8±5.3 | 136.2±9.2 | 267.8±12.9 | 426.3±23.4 | 532.2±21.6 | 616.3±27.7 | 677.5±29.2 | 715.8±29.7 |
| **FL** | **20-40cm** | 23.3±2.2 | 61.4±3.9 | 121.4±7.8 | 185.4±11.6 | 231.1±14.2 | 269.1±14.7 | 295±13.7 | 311.4±15.3 |
| **FL** | **40-60cm** | 16.6±2.3 | 43.6±3.2 | 85.6±6.5 | 127.3±10.5 | 155.7±12.1 | 180.0±13.5 | 198.8±14.0 | 209.6±15.2 |
| **FL** | **60-80cm** | 11.9±1.4 | 30.7±3.5 | 59.3±5.9 | 88.9±6.7 | 109.1±9.0 | 126.7±9.7 | 138.5±10.5 | 145.3±11.4 |
| **FL** | **80-100cm** | 8.8±0.4 | 23.5±1.7 | 47.0±2.1 | 71.5±3.1 | 86.0±4.1 | 99.4±4.6 | 108.7±5.1 | 113.7±5.2 |
| **AL** | **0-20cm** | 73.8±4.1 | 190.8±12.7 | 371.5±24.8 | 586.4±35.8 | 757.2±47.0 | 885.2±49.0 | 975.3±55.4 | 1043.1±55.9 |
| **AL** | **20-40cm** | 39.1±4.7 | 101.7±7.9 | 195.6±10.4 | 300.8±12.4 | 387.7±22.0 | 454.7±31.9 | 498.7±37.4 | 535.3±38.9 |
| **AL** | **40-60cm** | 26.4±1.0 | 67.6±4.4 | 131.8±8.6 | 196.5±12.2 | 251.5±16.0 | 298.2±20.4 | 328.3±23.2 | 351.6±24.4 |
| **AL** | **60-80cm** | 20.5±1.4 | 50.7±3.9 | 97.9±6.5 | 147.4±7.6 | 188.5±10.0 | 220.5±10.3 | 242.2±11.1 | 259.1±12.0 |
| **AL** | **80-100cm** | 17.9±1.8 | 45.2±4.9 | 83.3±9.8 | 122.9±13.5 | 152.9±14.9 | 179.4±16.5 | 198.2±18.0 | 210.7±16.7 |

**S2. *C*_min_ and SOC mineralization efficiency from each land-use types over the 42-day study (Mean ± SD)**

| **Code** | **Soil depth** | **Cmin (mg kg^-1^)** | **SOC mineralization efficiency (%)** | **Code** | **Soil depth** | **Cmin (mg kg^-1^)** | **SOC mineralization efficiency (%)** |
| --- | --- | --- | --- | --- | --- | --- | --- |
| **ZB28** | **0-20cm** | 988.9±48.6 | 4.91±0.04 | **ZB8** | **0-20cm** | 758.3±35.3 | 4.69±0.40 |
| **ZB28** | **20-40cm** | 506.1±27.3 | 3.33±0.19 | **ZB8** | **20-40cm** | 356.0±18.9 | 3.05±0.34 |
| **ZB28** | **40-60cm** | 324.9±19.2 | 2.80±0.35 | **ZB8** | **40-60cm** | 232.8±9.4 | 2.72±0.26 |
| **ZB28** | **60-80cm** | 242.1±16.9 | 2.49±0.22 | **ZB8** | **60-80cm** | 163.4±8.0 | 2.33±0.05 |
| **ZB28** | **80-100cm** | 190.4±16.2 | 2.25±0.22 | **ZB8** | **80-100cm** | 126.4±9.3 | 2.07±0.07 |
| **ZB20** | **0-20cm** | 923.2±50.7 | 4.86±0.53 | **FL** | **0-20cm** | 715.8±29.7 | 4.66±0.48 |
| **ZB20** | **20-40cm** | 461.5±24.3 | 3.26±0.11 | **FL** | **20-40cm** | 311.4±15.3 | 2.90±0.07 |
| **ZB20** | **40-60cm** | 288.4±14.7 | 2.75±0.3 | **FL** | **40-60cm** | 209.6±15.2 | 2.61±0.07 |
| **ZB20** | **60-80cm** | 202.9±13.0 | 2.44±0.23 | **FL** | **60-80cm** | 145.3±11.4 | 2.19±0.08 |
| **ZB20** | **80-100cm** | 160.8±6.8 | 2.14±0.23 | **FL** | **80-100cm** | 113.7±5.2 | 1.98±0.05 |
| **ZB15** | **0-20cm** | 815.0±47.0 | 4.75±0.37 | **AL** | **0-20cm** | 1043.1±55.9 | 4.97±0.17 |
| **ZB15** | **20-40cm** | 402.9±22.7 | 3.05±0.06 | **AL** | **20-40cm** | 535.3±38.9 | 3.38±0.44 |
| **ZB15** | **40-60cm** | 252.5±17.0 | 2.72±0.19 | **AL** | **40-60cm** | 351.6±24.4 | 2.83±0.30 |
| **ZB15** | **60-80cm** | 176.2±8.0 | 2.37±0.11 | **AL** | **60-80cm** | 259.1±12.0 | 2.50±0.24 |
| **ZB15** | **80-100cm** | 141.8±10.8 | 2.10±0.37 | **AL** | **80-100cm** | 210.7±16.7 | 2.28±0.21 |

**S3. SOC, TON, C:N, pH, URE, INV, EOC, MBC and MBC/SOC of soil before SOC mineralization (Mean ± SD)**

| **Code** | **Soil depth** | **SOC (g/kg)** | **TON (g/kg)** | **C:N** | **pH** | **URE (mg/g)** | **INV (mg/g)** | **EOC(g/kg)** | **MBC(mg/kg)** | **MBC/SOC(%)** |
| --- | --- | --- | --- | --- | --- | --- | --- | --- | --- | --- |
| **ZB28** | **0-20cm** | 20.13±0.86 | 2.86±0.2 | 7.04±0.2 | 7.2±0.3 | 0.43±0.02 | 12.14±0.54 | 2.81±0.05 | 601.72±33.27 | 2.99±0.15 |
| **ZB28** | **20-40cm** | 15.23±0.59 | 2.21±0.11 | 6.9±0.52 | 7.35±0.23 | 0.36±0.02 | 9.84±0.55 | 2.07±0.08 | 397.34±26.31 | 2.62±0.27 |
| **ZB28** | **40-60cm** | 11.67±0.79 | 1.8±0.07 | 6.47±0.19 | 7.4±0.24 | 0.3±0.01 | 9.38±0.66 | 1.53±0.09 | 271.64±18.77 | 2.34±0.32 |
| **ZB28** | **60-80cm** | 9.76±0.72 | 1.55±0.07 | 6.29±0.53 | 7.43±0.25 | 0.26±0.01 | 8.75±0.41 | 1.24±0.1 | 205.19±9.97 | 2.11±0.26 |
| **ZB28** | **80-100cm** | 8.49±0.66 | 1.39±0.09 | 6.1±0.41 | 7.45±0.23 | 0.13±0.01 | 8.28±0.39 | 1.04±0.03 | 174.04±13.41 | 2.06±0.31 |
| **ZB20** | **0-20cm** | 19.08±1.15 | 2.79±0.12 | 6.86±0.43 | 7.27±0.23 | 0.36±0.02 | 11.94±0.84 | 2.58±0.18 | 547.12±40.91 | 2.88±0.37 |
| **ZB20** | **20-40cm** | 14.18±0.6 | 2.15±0.14 | 6.62±0.5 | 7.37±0.28 | 0.3±0.01 | 9.58±0.7 | 1.86±0.17 | 353.98±25.65 | 2.5±0.08 |
| **ZB20** | **40-60cm** | 10.54±0.75 | 1.68±0.11 | 6.28±0.37 | 7.43±0.25 | 0.24±0.01 | 8.94±0.65 | 1.32±0.08 | 240.41±12.12 | 2.29±0.25 |
| **ZB20** | **60-80cm** | 8.33±0.65 | 1.37±0.1 | 6.08±0.22 | 7.46±0.27 | 0.19±0.01 | 8.1±0.49 | 1.02±0.13 | 166.63±10.86 | 2.01±0.17 |
| **ZB20** | **80-100cm** | 7.54±0.69 | 1.25±0.08 | 6.04±0.31 | 7.51±0.25 | 0.11±0 | 7.45±0.51 | 0.88±0.02 | 144.46±10.71 | 1.92±0.19 |
| **ZB15** | **0-20cm** | 17.23±1.49 | 2.6±0.11 | 6.63±0.48 | 7.29±0.22 | 0.32±0.03 | 11.23±0.48 | 2.29±0.38 | 472.12±22.73 | 2.75±0.26 |
| **ZB15** | **20-40cm** | 13.19±0.82 | 2.06±0.17 | 6.4±0.23 | 7.41±0.25 | 0.28±0.01 | 8.8±0.5 | 1.68±0.03 | 310.72±17.23 | 2.36±0.02 |
| **ZB15** | **40-60cm** | 9.3±0.44 | 1.54±0.04 | 6.06±0.25 | 7.45±0.24 | 0.21±0.02 | 8.49±0.41 | 1.14±0.11 | 198.9±10.02 | 2.14±0.15 |
| **ZB15** | **60-80cm** | 7.43±0.26 | 1.28±0.07 | 5.84±0.53 | 7.5±0.36 | 0.16±0.01 | 7.26±0.32 | 0.88±0.09 | 144.69±6.07 | 1.95±0.07 |
| **ZB15** | **80-100cm** | 6.86±0.81 | 1.19±0.08 | 5.73±0.32 | 7.52±0.32 | 0.1±0.01 | 6.91±0.28 | 0.77±0.11 | 124.79±8.62 | 1.84±0.26 |
| **ZB8** | **0-20cm** | 16.23±1.15 | 2.55±0.17 | 6.38±0.41 | 7.33±0.23 | 0.25±0.01 | 10.86±0.46 | 2.07±0.11 | 415.56±20.01 | 2.57±0.26 |
| **ZB8** | **20-40cm** | 11.71±0.73 | 1.94±0.09 | 6.06±0.56 | 7.45±0.16 | 0.21±0.01 | 7.81±0.56 | 1.44±0.13 | 253.58±14.06 | 2.18±0.26 |
| **ZB8** | **40-60cm** | 8.59±0.54 | 1.48±0.08 | 5.84±0.7 | 7.48±0.26 | 0.18±0.01 | 6.42±0.42 | 1.02±0.13 | 168.84±8.51 | 1.97±0.19 |
| **ZB8** | **60-80cm** | 7.02±0.5 | 1.23±0.07 | 5.7±0.45 | 7.53±0.25 | 0.13±0.01 | 5.94±0.42 | 0.79±0.1 | 132.38±5.78 | 1.89±0.05 |
| **ZB8** | **80-100cm** | 6.11±0.31 | 1.11±0.08 | 5.54±0.68 | 7.55±0.18 | 0.1±0.01 | 4.03±0.24 | 0.67±0.04 | 106.66±7.75 | 1.74±0.06 |
| **FL** | **0-20cm** | 15.42±0.94 | 2.51±0.16 | 6.15±0.47 | 7.42±0.26 | 0.18±0.01 | 10.12±0.43 | 1.91±0.13 | 353.82±16.67 | 2.3±0.2 |
| **FL** | **20-40cm** | 10.75±0.73 | 1.83±0.1 | 5.88±0.15 | 7.47±0.35 | 0.15±0.02 | 6.55±0.29 | 1.27±0.12 | 220.38±10.76 | 2.06±0.22 |
| **FL** | **40-60cm** | 8.02±0.59 | 1.39±0.06 | 5.78±0.51 | 7.54±0.23 | 0.12±0.01 | 4.63±0.39 | 0.92±0.05 | 149.5±6.71 | 1.87±0.06 |
| **FL** | **60-80cm** | 6.63±0.47 | 1.19±0.05 | 5.57±0.6 | 7.55±0.26 | 0.1±0.01 | 3.38±0.32 | 0.73±0.04 | 113.86±6.76 | 1.72±0.04 |
| **FL** | **80-100cm** | 5.75±0.23 | 1.06±0.09 | 5.42±0.32 | 7.58±0.23 | 0.08±0.01 | 2.95±0.25 | 0.62±0.05 | 92.66±6.62 | 1.62±0.18 |
| **AL** | **0-20cm** | 21.01±0.96 | 2.91±0.16 | 7.22±0.08 | 7.17±0.16 | 0.53±0.04 | 13.99±0.65 | 3.08±0.18 | 651.09±27.19 | 3.1±0.21 |
| **AL** | **20-40cm** | 15.9±1.03 | 2.27±0.14 | 7.02±0.05 | 7.28±0.19 | 0.5±0.02 | 10.55±0.65 | 2.24±0.19 | 447.29±21.3 | 2.82±0.29 |
| **AL** | **40-60cm** | 12.46±0.83 | 1.87±0.09 | 6.67±0.49 | 7.38±0.17 | 0.45±0.03 | 10.15±0.45 | 1.72±0.04 | 309.84±19.43 | 2.49±0.21 |
| **AL** | **60-80cm** | 10.39±0.53 | 1.61±0.09 | 6.46±0.38 | 7.41±0.22 | 0.37±0.03 | 9.46±0.76 | 1.4±0.13 | 242.99±13.89 | 2.34±0.19 |
| **AL** | **80-100cm** | 9.25±0.64 | 1.47±0.09 | 6.3±0.37 | 7.42±0.18 | 0.15±0.01 | 9.03±0.8 | 1.2±0.07 | 201.21±9.06 | 2.18±0.15 |
